# Evaluation of a remote monitoring system in people with mental illness and medical co-morbidity

**Published:** 2012-06-15

**Authors:** Sarah Ilana Pratt

**Affiliations:** Dartmouth Medical School, USA

**Keywords:** mental illness, telehealth, medical comorbidity, remote monitoring

## Abstract

**Introduction:**

High medical co-morbidity, poor health behaviors, medication side effects, and inadequate health care contribute to a 25–30 year disparity in life expectancy between people with serious mental illness (SMI) and people without mental illness. There is an urgent need to enable providers to monitor health status and risk daily to improve health outcomes for this high-risk, high cost group. Telehealth interventions hold promise for monitoring patients remotely, with the hope of reducing costs and increasing accessibility of interventions; however, they have rarely been used or evaluated in people with SMI.

**Aims and objectives:**

Primary aim: Determine the feasibility and acceptability of an in-home remote monitoring system programmed with daily dialogues specific to the user’s medical and psychiatric condition in outpatients diagnosed with SMI and either diabetes, hypertension, cardiac disease, COPD, or chronic pain.

Secondary aim: Evaluate the potential effectiveness of remote monitoring with respect to management of psychiatric and medical illness symptoms.

**Methods:**

Seventy community mental health center (CMHC) clients were randomly assigned to either immediately receive the remote monitoring system (n=37) for 6 months or to receive it after a 6-month wait (n=33). Service use, illness management and recovery, subjective and objective health, medical co-morbidity, disease management self-efficacy, and psychiatric symptoms were assessed at baseline and at 6 and 12 months. Satisfaction with the device was assessed after 6 months. Responses to daily dialogue questions were reviewed by a CMHC nurse care manager.

**Results:**

The telehealth device was highly acceptable and valued. Adherence with daily sessions was extremely high. Mean adherence across all participants for 6 months was 71%. Over half completed 89% or more of their sessions. Participants who immediately received the device demonstrated greater improvement in overall psychiatric symptom severity compared to the waitlist group. Use of the device resulted in significant reduction of diastolic blood pressure and a significant increase in depression self-management. Among individuals with diabetes, remote monitoring resulted in lower fasting glucose and lower use of urgent care visits. Sixty-six percent of diabetic participants had glucose >140 at baseline. After using the device for 3 months, 38% had a >20% reduction in glucose, and 14% reduced their glucose by >100. At 6 months, 50% had achieved a >20% reduction. Mean glucose dropped from 209 at baseline to 128 at 6 months. Satisfaction with the device at 6 months was extremely high; 81% reported that they would be very willing to continue using it.

**Conclusions:**

In the USA, 1300 community mental health centers annually serve 6 million adults, children, and families. Facilitating patient/clinician communication is particularly important in SMI treatment due to common barriers, specifically problem behaviors and poor adherence. If use of remote monitoring with psychiatric patients can improve symptom management, support people’s ability to live more independently, and reduce emergency room visits, this has great potential to improve outcomes and quality of life. The findings here that remote monitoring led to improved management of psychiatric symptoms and health outcomes, especially in patients with SMI and diabetes, therefore have international implications.

